# Management of uterine artery pseudoaneurysm: a case report of noninterventional treatment with systematic review

**DOI:** 10.1016/j.xagr.2025.100555

**Published:** 2025-08-05

**Authors:** Vincent Landré, Hans-Christoph Pape, Ksenija Slankamenac, Nicole Ochsenbein-Kölble, Nina Kimmich

**Affiliations:** 1 Department of Obstetrics, University Hospital Zurich, University of Zurich, Zurich, Switzerland (Ochsenbein-Kölble and Kimmich); 2 Department of Traumatology, University Hospital Zurich, University of Zurich, Zurich, Switzerland (Landré and Pape); 3 Institute of Emergency Medicine, University Hospital Zurich, University of Zurich, Zurich, Switzerland (Slankamenac)

**Keywords:** blood vessel rupture, noninterventional management, pelvic vessels, pregnancy, uterine artery, uterine pseudoaneurysm

## Abstract

**Background:**

Uterine artery pseudoaneurysm (UAP) is a rare but potentially life-threatening condition that can result in severe hemorrhage. Due to its nonspecific clinical presentation, it is often misdiagnosed, leading to delays in appropriate intervention. UAP commonly arises following uterine trauma, including cesarean section, vaginal delivery, and other gynecological procedures or pathologies, such as endometriosis. While selective arterial embolization is the preferred treatment, noninterventional management may be a viable alternative in selected cases.

**Case Report:**

We present a case of a 33-year-old woman in her first pregnancy diagnosed with UAP at 27 gestational weeks (GW). She complained about intermittent left lower abdominal pain without vaginal bleeding. Initial imaging with Doppler ultrasonography and noncontrast magnetic resonance imaging (MRI) identified a left paracervical mass consistent with a UAP. Further imaging with contrast-enhanced MRI confirmed the diagnosis and revealed thrombosis of the lesion. Given the absence of perfusion and clinical stability, a noninterventional approach was pursued. The patient remained hemodynamically stable and was discharged after 6 days of hospitalization. At 38+4 GW, she underwent a scheduled cesarean section, and both maternal and neonatal outcomes were favorable. Follow-up at 12 months postdiagnosis showed no recurrence or complications.

**Methods:**

A systematic review was conducted, analyzing peer-reviewed studies from 1955 to 2024 in PubMed and EMBASE databases. Inclusion criteria focused on human studies reporting UAP, with data extracted on risk factors, diagnostic modalities, treatment strategies, and clinical outcomes. Statistical analyses included the Student’s *t* test for continuous variables and the Pearson chi-square test for categorical variables.

**Results:**

Out of 790 initially identified articles, 131 met inclusion criteria, comprising 144 patients with uterine artery UAP. Among these, 20 patients were pregnant, and 124 were nonpregnant. Comorbidities were more common in pregnant patients (55% vs 34.7%). Prior uterine manipulation occurred in 50% of pregnant and 90.3% of nonpregnant cases, with laparotomy and cesarean sections being most frequent. Vaginal bleeding was the most common symptom in nonpregnant patients (81.5%), while pain dominated in pregnant cases (85%). Imaging primarily involved ultrasound and angiography, combined with computed tomography (CT) in nonpregnant women (70% vs 35%) and MRI in pregnancy (70% vs 11.3%). Embolization was the main treatment (90% in pregnancy, 99% in nonpregnant), with few complications and no reported deaths. Statistical analysis showed a significant association in nonpregnant patients between vaginal bleeding and the need for transfusion (*P*<.05), as well as between bleeding and smaller UAP size (24.5 vs 32.3 mm, *P*<.05).

**Conclusion:**

UAP is rare and potentially serious. Vaginal bleeding is the most common presentation in nonpregnant patients, while pain is more frequent in pregnancy. Smaller UAPs were more likely to bleed in nonpregnant patients, suggesting rupture risk isn’t solely size-dependent. Diagnostics can be performed by ultrasound, angiography, and CT, or in pregnancy, especially by MRI. Embolization is highly effective and remains the standard of care. Noninterventional management may be cautiously considered in hemodynamically stable patients with spontaneously thrombosed or nonperfused UAPs, though evidence of its effectiveness remains limited. An individualized, multidisciplinary management remains the key. Further data collection will help refine treatment strategies.

**AJOG Global Reports at a Glance**

**Why was this study conducted?**

Uterine artery pseudoaneurysm (UAP) is rare but potentially life-threatening. Nonspecific presentation often delays diagnosis and treatment. Data on UAP in pregnancy, including management alternatives, remain limited

**Key findings**

Case: Spontaneous thrombosis of UAP at 27 weeks, successfully managed non-interventionally with favorable maternal and neonatal outcomes. Systematic review: Pain predominates in pregnancy, bleeding in nonpregnant patients; embolization effective in >90% of cases. Smaller UAPs more likely to rupture in nonpregnant patients.

**What does this add to what is known?**

Demonstrates feasibility of conservative management in selected stable pregnancies. Defines differences in presentation, imaging, and outcomes between pregnant and nonpregnant patients. Supports individualized, multidisciplinary care for optimal safety.

## Introduction

A uterine artery pseudoaneurysm (UAP) is a confined blood collection outside a vessel, maintaining communication with the arterial lumen due to a defect in the vessel wall.^1^ Uterine artery UAP is a rare, yet potentially life-threatening condition that can cause severe hemorrhage if not promptly diagnosed. UAP typically results from trauma to the uterine artery, commonly following procedures such as cesarean section, vaginal delivery, myomectomy, hysterectomy, or cervical dilation and curettage.^2^ It forms when trauma disrupts the arterial wall, leading to structural fragility. The estimated incidence is 3 to 6 cases per 1,000 deliveries, with approximately 40% arising after nontraumatic deliveries or abortions.^3^ Risk factors include previous uterine surgeries, infections, connective tissue disorders, and coagulopathies, all of which can predispose patients to vascular injury.^4^ UAP is most commonly diagnosed in the second and third trimesters, during labor, or in the early postpartum phase.^5^ This condition carries significant morbidity and mortality risks, particularly given the physiological increase in cardiac output during pregnancy, which peaks in the early third trimester at approximately 1.5 L/min—31% higher than prepregnancy levels.^6^ UAP is implicated in 0.3% to 1% of postpartum hemorrhage cases,^7^ and prompt diagnosis is crucial, as these vascular ruptures significantly elevate the risk of maternal complications.^8^ Doppler ultrasonography remains the primary diagnostic tool, typically revealing an anechoic or hypoechoic intravascular mass with turbulent arterial flow.^9^ Given the high risk of rupture, which may lead to maternal hypovolemic shock and fetal mortality in pregnant cases, the diagnosis of UAP requires urgent, multidisciplinary management. The preferred treatment is selective arterial embolization of the affected uterine artery branch, performed by an interventional radiologist, as it offers a favorable risk-benefit profile.^10^ Other options include open surgical ligation, percutaneous thrombin injection, and complete uterine artery embolization.^11^ In selected cases, noninterventional management may be considered, though evidence regarding its efficacy remains limited. This article presents a case of UAP in pregnancy managed noninterventionally, accompanied by a systematic review of current literature. Through synthesizing existing evidence, we aim to evaluate the effectiveness and safety of various management approaches, identify factors influencing treatment decisions, and assess clinical outcomes to achieve optimal patient care.

## Material and methods

First, we present a case of noninterventional treatment of UAP during pregnancy. Second, we performed a systematic review of peer-reviewed articles between 1955 and 2024 was conducted. Studies were included if they involved human subjects, reported on UAPs of the uterine artery, were published in the specified timeframe, and were written in English, French, or German. We excluded meta-analyses, systematic reviews, books, correspondence, conference abstracts, expert opinions, editorials, and in vitro/vivo studies. The primary databases for this search were PubMed and EMBASE, with the search being carried out from October 1 to November 1, 2024. Studies were reviewed against predefined inclusion criteria, and quantitative data on risk factors, diagnostic methods, treatment approaches, and outcomes were extracted. A full-text review of the included articles was performed to identify relevant information. All data were analyzed using SPSS Statistics (Version 23, IBM, Armonk, New York, USA) and initially evaluated descriptively. If the parameters followed a normal distribution, a Student’s *t* test was applied. For categorical data, the Pearson chi-square test was used. Statistical results were considered significant if the *P* value, based on a 95% confidence interval, was less than or equal to 0.05.

## Case report

**Figure 1:**
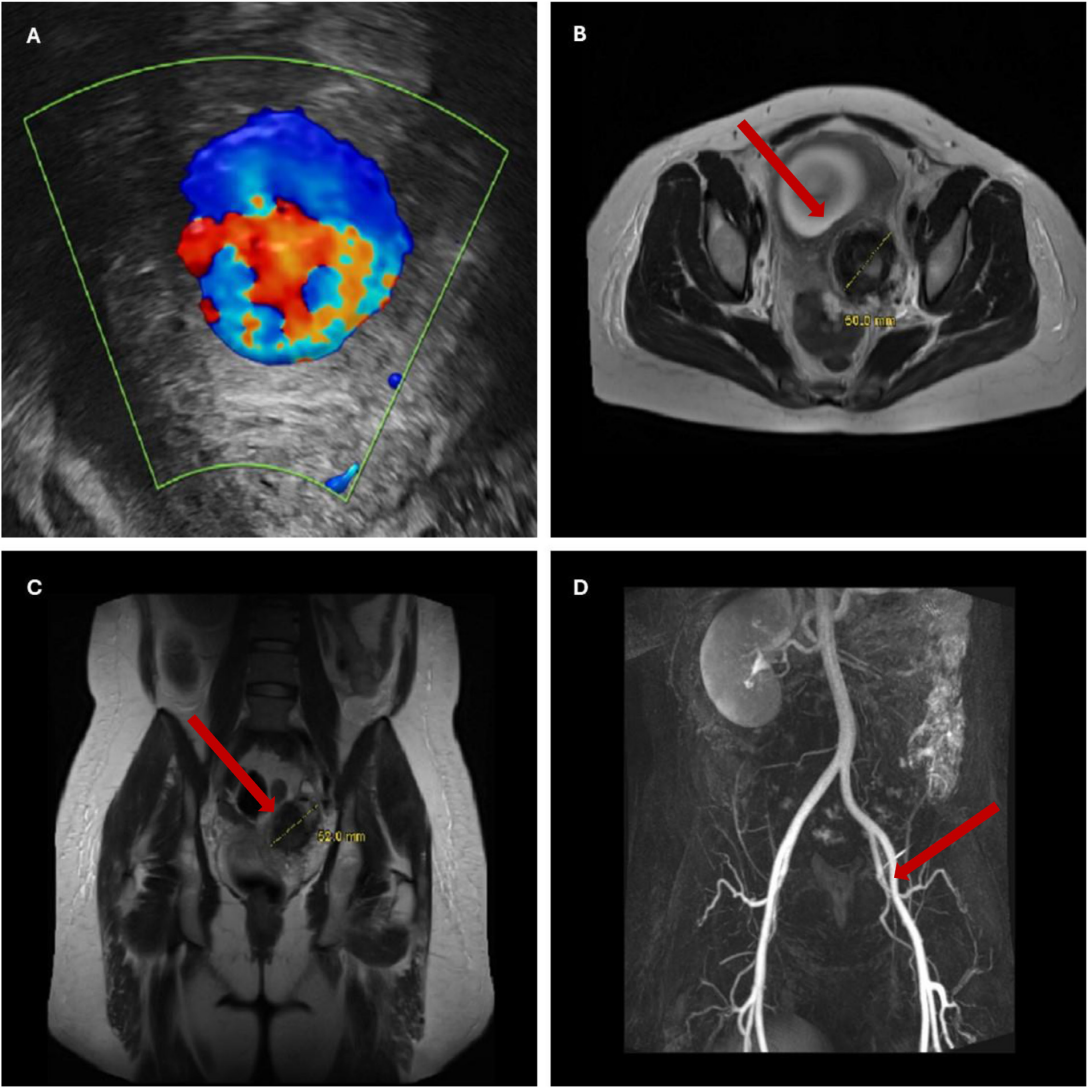
Diagnostics of the UAP. (A) The pathognomonic yin-yang sign showing the blood turbulence within the UAP of the left uterine artery in Doppler ultrasonography (GE Voluson E10 System, GE Healthcare, Zipf, Austria). (B) T2-weighted axial MRI showing a well-defined UAP. (C) T2-weighted coronal MRI showing the UAP. (D) Magnetic resonance angiography of the thrombosed UAP.

A 33-year-old woman in her first pregnancy presented at 27 gestational weeks (GW) with a 4-day history of intermittent left lower abdominal pain. The pain progressively worsened, accompanied by nausea and a single episode of vomiting. The patient’s physical examination was unremarkable, and initial laboratory tests, including urine analysis and genital swabs, revealed no signs of infection. Her medical history included endometriosis, a status postlaparoscopic nephrectomy for hydronephrosis in 2013, and a known penicillin allergy. She was taking low-dose aspirin, folic acid, electrolytes, and omega-3 supplements. Routine obstetric checks had been unremarkable, with normal blood pressure and fetal heart rate. Upon admission, vital signs were stable: blood pressure 125/65 mmHg, pulse 62 beats per minute, and temperature 36.5°C. Laboratory findings showed an elevated white blood cell count (23.96 G/L), though other parameters were within normal limits. An initial Doppler ultrasound revealed a 5 cm mass in the left paracervical region, highly suggestive of a UAP. The mass exerted a mild displacement of the cervix to the right. Noncontrast magnetic resonance imaging (MRI) revealed an ill-defined mass in the left paracervical area, with no clear connection to the uterine artery, yet characteristic features of a UAP. The patient was admitted for further observation, analgesia, and management of uterine contractions with nifedipine. Magnesium sulfate was administered for fetal neuroprotection, and corticosteroids were given to promote fetal lung maturity, in order to prepare for threatening preterm birth. Over the course of hospitalization, the patient’s symptoms gradually improved, and she transitioned to oral analgesia. Further imaging with contrast-enhanced MRI, performed 6 hours after the initial diagnosis, did not show enhancement, which confirmed a thrombosed UAP. Given the lack of perfusion and the absence of clinical progression, a noninterventional approach was adopted, with no need for surgical intervention or embolization. The decision was based on the absence of flow within the lesion and the stable clinical condition of the patient. This scenario is exceptional and does not represent a broadly applicable treatment pathway. The patient was closely monitored for any signs of deterioration, including recurrent pain, uterine contractions, or decreased fetal movements. Fetal heart rate and amniotic fluid levels always remained normal. The patient was discharged after 6 days in a stable condition. At 38+4 GW, she underwent a scheduled cesarean section due to the presence of the UAP. The surgery proceeded without complications, and a healthy male infant weighing 2830 grams was delivered. Twelve months postdiagnosis, the patient had no further symptoms related to the UAP, and her child was developing normally (Figure 1).

Landré. Management of uterine artery pseudoaneurysm. AJOG Glob Rep 2025*.*

## Systematic review and results

**Figure 2:**
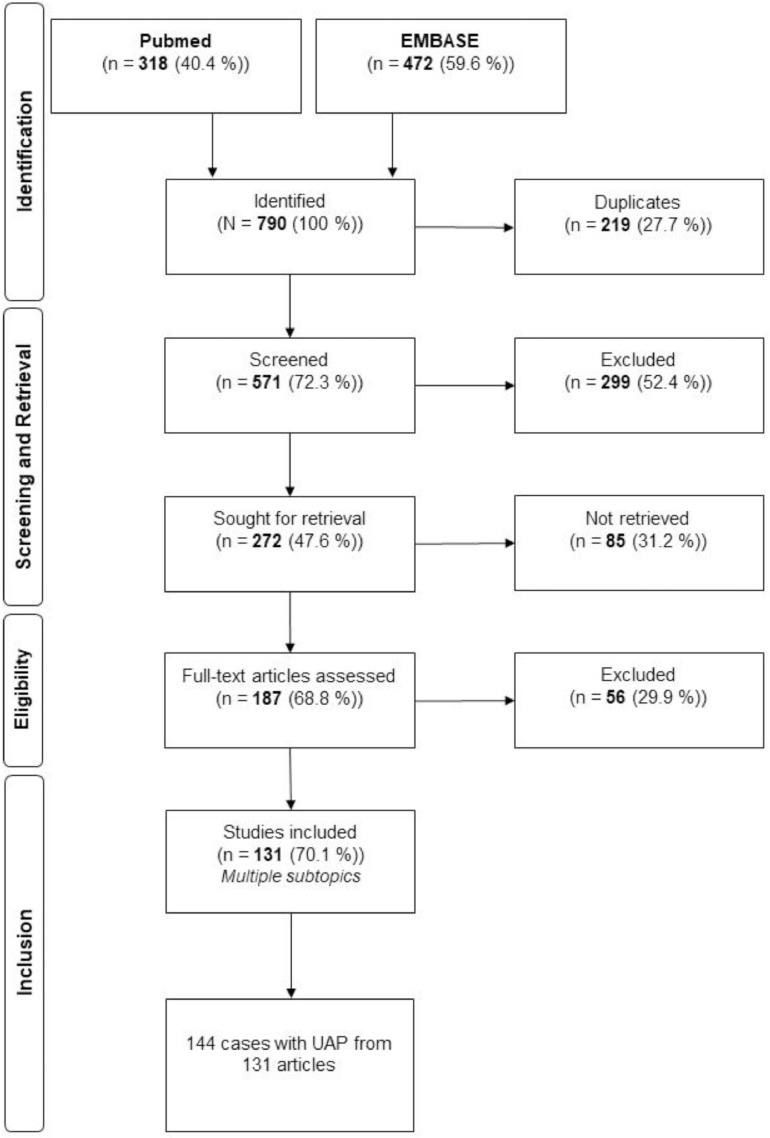
Flowchart of the literature analysis.

A thorough search utilizing MeSH terms initially retrieved 790 articles. Following the removal of 219 duplicates, 571 articles underwent screening, of which 272 met the inclusion criteria. Ultimately, 131 articles were selected for analysis (Figure 2). The study encompassed a total of 144 patients from 131 articles diagnosed with a UAP. A document presenting the search strategy is attached as a supplement file.

Landré. Management of uterine artery pseudoaneurysm. AJOG Glob Rep 2025.

Table 1 presents demographic data and prior uterine manipulation in pregnant and nonpregnant women with UAP.

**Table 1:** Patients’ characteristics and previous history of uterine manipulation

Characteristics	Pregnant women (*n*=20)	Nonpregnant women (*n*=124)
**Age in years**^a^	32.75 (29–36.3)	32 (27.25–35.75)
**Comorbidities**	17 in 11 cases (55%)	71 in 58 cases (34.7%)
Endometriosis	5 (25%)	5 (4%)
Miscarriages	3 (15%)	22 (17.7%)
*N*=1	1 (5%)	20 (16.1%)
*N*=2	2 (10%)	2 (1.6%)
Ovarian cysts	3 (15%)	5 (4%)
Arterial hypertension	2 (10%)	1 (0.8%)
Appendicitis acuta	1 (5%)	1 (0.8%)
Cervical dysplasia	1 (5%)	1 (0.8%)
Hypercoagulability	1 (5%)	0
Myoma	1 (5%)	11 (8.9%)
Arteriovenous malformation	0	1 (0.8%)
Von Willebrand disease	0	3 (2.4%)
Trophoblastic disease	0	3 (2.4%)
Diabetes mellitus	0	1 (0.8%)
Uterus dysplasia	0	1 (0.8%)
Pulmonary embolism	0	1 (0.8%)
Pulmonary hypertension	0	1 (0.8%)
Rheumatic heart disease	0	1 (0.8%)
HELLP syndrome	0	2 (1.6%)
Chorioamnionitis	0	2 (1.6%)
Carcinoma	0	7 (5.6%)
Substance abuse	0	1 (0.8%)
Ehlers Danlos syndrome	0	1 (0.8%)
Arterial fibrillation	0	1 (0.8%)
Cavernous hemangioma	0	1 (0.8%)
**Any manipulation**	10 (50%)	112 (90.3)
Laparoscopy	5 (25%)	19 (15.3%)
In vitro fertilization (IVF)	3 (15%)	5 (4%)
Laparotomy	2 (10%)	74 (60%)
Cesarean section	1 (5%)	70 (56.5)
Curettage	1 (5%)	23 (18.5)
Surgical or medical abortion	1 (5%)	5 (4%)

a Median (25th–75th percentile).

Table 2 shows the initial symptoms at referral and the methods performed for diagnosing UAP.

**Table 2:** Symptoms and diagnostics

Symptoms and diagnostics	Pregnant women (*n*=20)	Nonpregnant women (*n*=124)
Vaginal bleeding	5 (25%)	101 (81.5%)
Cardiovascular shock	0	24 (19.4%)
Abdominal pain	17 (85%)	32 (25.8%)
Fever	0	10 (8.1%)
Amenorrhea	0	2 (1.6%)
Asymptomatic	2 (10%)	10 (8.1%)
Ultrasonography	16 (80%)	118 (95.2%)
Angiography	18 (90%)	115 (92.7%)
CT	7 (35%)	78 (70.2)
MRI	14 (70%)	14 (11.3)

Multiple UAPs were observed in 2 pregnant patients (10%) and 5 nonpregnant patients (4%). In the pregnant group, 80% of the UAPs were located on the left side (*n*=16) and 20% on the right side (*n*=4). Among nonpregnant patients, 66.1% of UAPs were on the left (*n*=80), while 33.9% were on the right (*n*=41). The average UAP diameter was slightly larger in pregnant patients with at 28.8 mm±18.4 mm, compared to 26.1 mm±15.2 mm in the nonpregnant group. The performed treatment modalities of UAP are presented in Table 3. A subset of 11 patients received two or more therapeutic interventions for the management UAP. Specifically, five patients underwent a combination of noninterventional management followed by an embolization due to persistence of the UAP. One of these patients also required diagnostic laparoscopy. In six cases, embolization was combined or followed by laparotomy due to insufficient closure of the UAP. One patient underwent a percutaneous injection therapy followed by laparotomy due to insufficient coagulation of the UAP.

**Table 3:** Treatment modalities of UAP

Therapy	Pregnant women (*n*=20)	Nonpregnant women (*n*=124)
**Primary treatments**		
Hysterectomy by laparotomy	0	5 (4%)
Open surgery via laparoscopy	0	1 (1%)
Percutaneous thrombin injection	0	1 (1%)
Embolization	19 (95%)	112 (90%)
Noninterventional	1 (5%)	5 (4%)
**Revision due to persistent UAP**	2 (10%)	11 (9%)
Embolization	2 (10%)	8 (6%)
Open surgery via laparoscopy	0	3 (3%)
**Supportive treatment**		
Blood transfusion	6 (30%)	48 (39%)
Antibiotics	3 (15%)	22 (18%)

Overall, complication rates were low among both pregnant and nonpregnant patients with UAP. In the pregnant group, one case (5%) of eclampsia was reported, with no instances of re-bleeding, infection, pulmonary embolism, or death. In contrast, complications in the nonpregnant subgroup included re-bleeding in seven patients (5.6%), infections in three (2.4%), and one case of pulmonary embolism (0.8%). Importantly, no fatalities were reported in either group.

Using the Chi-square test for hypothesis testing, no statistically significant association was found between the presenting symptom at the time of diagnosis and the occurrence of shock or the need for laparotomy as treatment, in either the pregnant or nonpregnant subgroups. However, in the nonpregnant subgroup, there was a statistically significant association between the presence of vaginal bleeding and the requirement for blood product transfusion (*P*<.05). No significant association was observed between the type of therapy administered and the occurrence of complications.

A statistically significant association was identified in the nonpregnant subgroup between the presence of vaginal bleeding and UAP diameter (*P*<.05). Specifically, the mean diameter was 24.5 mm in patients with vaginal bleeding, compared to 32.3 mm in those without. No statistically significant association was found between lesion diameter and the occurrence of shock, the need for blood product transfusion, or postoperative complications in either the pregnant or nonpregnant subgroup.

## Discussion

UAP is a rare but potentially life-threatening vascular lesion initially described in 1994.^3^ It can cause threatening hemorrhage and therefore needs to be diagnosed early and treated by a multidisciplinary team to reduce morbidity and mortality.^12^ This study presents a unique case of noninterventional managed UAP during pregnancy and offers a comprehensive review of the literature to contextualize current diagnostic and therapeutic approaches. We emphasize the importance of considering UAP in the differential diagnosis of atypical abdominal pain during pregnancy, even in the absence of vaginal bleeding or hemodynamic instability. For the detection of UAP, Doppler ultrasonography, MRI, computed tomography, and angiography are utilized.^12^ Although embolization remains the standard treatment for UAP, especially in postpartum and nonpregnant cases due to its effectiveness in rapid bleeding control, our review highlights a noninterventional management approach in hemodynamically stable patients with thrombosed or nonperfused lesions. Out of 20 reported pregnancy-associated UAP cases, 95% underwent embolization, whereas only our patient was managed without a surgical intervention. While our case demonstrates that “watchful waiting” may be feasible in highly selected cases, this outcome should be interpreted cautiously. The systematic review highlights that noninterventional management is rarely successful and appears effective primarily when the UAP is spontaneously thrombosed and the patient is clinically stable.^13^ The systematic review confirmed that vaginal bleeding remains the most common presenting symptom, particularly in the nonpregnant group (81.5%), while pain was more prevalent among pregnant patients (85%). Vaginal bleeding was significantly associated with a need for blood transfusion (*P*<.05), aligning with previous findings that associate vaginal hemorrhage with greater clinical intervention.^12^ However, no statistically significant association was found between presenting symptoms and the need for laparotomy or the occurrence of complications, which suggests that symptom severity may not always be predictive of adverse outcomes. Interestingly, lesion size did not correlate with shock, transfusion requirements, or complications in either group, although in the nonpregnant population bleeding was more likely in patients with smaller UAPs (mean diameter 24.5 vs 32.3 mm, *P*=.017). This counterintuitive finding may reflect variability in lesion morphology or rupture dynamics rather than size alone and warrants further investigation.

Despite the broad application of embolization, our findings suggest that individualized treatment planning is crucial—particularly in pregnant patients, where radiation exposure and the risks of invasive procedures must be weighed against the potential for spontaneous resolution.^14^ MRI, including contrast-enhanced sequences, were particularly useful in our case for assessing lesion perfusion and guiding decision-making without the need for ionizing radiation. However, its limited availability and cost may pose challenges in less-resourced settings.^15^^,^^16^ From a clinical management perspective, this case reinforces the value of a multidisciplinary approach involving obstetrics, radiology, and trauma surgery. The absence of complications or recurrence after 1 year supports the long-term safety of noninterventional treatment in selected cases.

Limitations of this study include the retrospective nature of the literature review and the inherent bias toward published cases, which may underreport uncomplicated or noninterventionally managed UAP. Furthermore, the rarity of UAP limits the ability to conduct robust prospective trials, emphasizing the need for international case registries to collect standardized data and inform future guidelines.

While selective arterial embolization remains the treatment of choice for UAP, particularly in actively bleeding or unstable patients, our findings suggest that noninterventional management may be cautiously considered in specific cases with thrombosed or nonperfused UAPs, but the rarity of such successful outcomes in the literature underscores that this is an exception rather than a rule. This approach requires accurate imaging, multidisciplinary monitoring, and careful patient selection. Continued research is essential to refine diagnostic criteria and management pathways, ensuring optimal outcomes while minimizing unnecessary interventions.

## CRediT authorship contribution statement

**Vincent Landré:** Writing – review & editing, Writing – original draft, Investigation, Data curation, Conceptualization. **Hans-Christoph Pape:** Supervision. **Ksenija Slankamenac:** Writing – review & editing, Supervision, Project administration, Formal analysis. **Nicole Ochsenbein-Kölble:** Writing – review & editing, Supervision, Funding acquisition. **Nina Kimmich:** Writing – review & editing, Resources, Project administration, Investigation, Funding acquisition, Conceptualization.
